# Existential Positive Psychology (EPP): A Positive Tool for Healing Existential Anxieties in South Africa during, and after, the COVID-19 Pandemic

**DOI:** 10.3390/ijerph191610248

**Published:** 2022-08-18

**Authors:** Kathryn Anne Nel, Saraswathie Govender

**Affiliations:** 1 School of Social Sciences, University of Limpopo, Polokwane 0727, South Africa; 2 Department of Psychology, University of Limpopo, Polokwane 0727, South Africa

**Keywords:** Afrocentricity, authentic, COVID-19, existence, humanity

## Abstract

Globally, humanity is in the grip of the COVID-19 pandemic; thus, we question our individual, and collective, behaviours. Long periods of lockdown and ever-escalating death rates have found people asking questions such as “What is the point of carrying on?” This is exacerbated by the world’s burgeoning ecological crisis. Humanity is beginning to wonder if it belongs on the planet when its footprint has caused such rampant destruction to forests, oceans, the animal kingdom, and other ecological entities. Existential positive psychology (EPP) seeks to uncover truths about humankind’s existence, survival, and, thus, meaning in life. We, as people, need to make sense of our reason for being as we struggle with our anxieties and seek to become authentic. This discussion paper contends that EPP can help humanity find the courage to challenge, and heal, its existential anxieties, namely, death, isolation, freedom, and meaningless, in order to find individual and group identities, as well as overall mental wellness (or happiness), specifically in a South African context, during the COVID-19 pandemic. The writings of Wong, who works within the framework of EPP, and those of Frankl, a holocaust survivor, whose work falls within the scope of humanistic and existential psychology and Asante’s Afrocentrism, which is a philosophical framework grounded on the African continent, are used to support this argument.

## 1. Introduction

Existential positive psychology (EPP) stems from existentialism. It looks at how humans experience their lives subjectively; thus, it is implicit to human existence. It has a positive theoretical standpoint, as it stresses how humanity, in spite of existential anxieties, has the courage to confront these challenges in order to live an authentic life, which infers mental wellness. Existential positive psychology emphasises the integration of positive and negative experiences and highlights the fact that it is only through meeting our challenges that we are able to “grow” psychologically [1]. In other words, we have to overcome our struggles in life and become resilient so that we can strengthen and develop humanity in a positive manner. Life and death are intertwined as the song goes, “you can’t have one without the other”!

That is why the mission of existential positive psychology is to investigate ways to reduce human suffering and transform it into human flourishing [2] (p. 2). Humanity suffers loss of meaning, lack of purpose, and suffering; nonetheless, through all of the destruction and damage, individuals must find themselves and understand the meaning of their lives in order to live authentically and to flourish. The concept of flourishing encompasses mental wellness and happiness, which are achieved through human relationships, love, meaning, and individual achievements [3]. In this paper, reference to humanity flourishing encompasses all of these notions. Meaning in life refers to the ability to ask questions of oneself in terms of what, why, and how goals are pursued, and do they facilitate finding individual and/or collective meaning in life so that individuals and communities can find meaning as a coherent whole. This is consistent with the concept of Ubuntu in Southern Africa; that is, I am because we are. In other words, we find meaning in life together [4].

Today, the world is still preoccupied by the COVID-19 pandemic, though it is now becoming thought of as endemic and something we must live with. This, together with the burgeoning ecological crisis or global warming, has resulted in an existential crisis for all humanity. Developing countries, such as South Africa, have many difficult challenges; most have crippled economies and social systems to say nothing of health systems that are teetering on the verge of collapse. These shortcomings have been highlighted by the global pandemic; however, if we use the lens of EPP, we can take the negatives and find the positives and turn our anxieties into our rationale for living and, thus, transform the failings of our world (internal and external) into an authentic rationale for human flourishing. Fundamentally, suffering is redemptive, as it promotes resilience through which we will find hope and happiness [2].

Firstly, we will provide a very brief overview of Wong, Frankl, and Asante’s concepts, followed by a discussion of how EPP can provide us with underpinnings to an authentic life (world) with reference to these authors (we encourage readers to delve into their works). Asante has an African worldview, and it may seem that he does not belong in a text on EPP; however, an African worldview is essential to “turning the world around”. This in terms of humanities mental and physical health is irrefutably linked to the wellbeing (both physical and mental) of all our peoples and the planet, our ancestral mother Gaia. Then, we look at how the theories can be integrated in terms of EPP, followed by how the pandemic has provoked our existential anxieties, and, lastly, how EPP theory and practice can help South Africans flourish through their suffering.

### 1.1. A Brief Overview of Key Points in Wong, Frankl, and Asante’s Theories

This section is included so that readers can appreciate how very different their lives were. Through their works, it can be inferred that they all found meaning in life, were resilient, and overcame existential anxieties in order to help humanity.

#### 1.1.1. Paul T.P. Wong—Existential Positive Psychology (EPP)

Wong’s name, we assert, has become synonymous with EPP. He has a background in learning theory, social cognition, and existential psychology before he integrated his knowledge in EPP. He combined Frankl’s existential psychology with positive psychology, which became known as existential positive psychology (EPP). In doing this, he recognised the need to incorporate and recognise cross-cultural psychology internationally into the ambit of EPP.

Existential psychology encapsulates an approach to human challenges; however, many existentialist writings tend to be caught up in the dark side of life [1]. This is his rationale for integrating existential and positive psychology (PP), as PP is underpinned by the positive. Additionally, Wong added questions about identity and happiness, as he wanted to see how human suffering could be transformed into human flourishing [5]. Existential positive psychology (EPP) thus looks at the human condition in terms of “Who am I?” and how can I find happiness? These questions relate to how an individual lives their lives and what choices they make to achieve worth because it is death that is humanity’s common denominator. Wong asserts that EPP extends the meaning of PP in terms of “resilience and positive change” [6] (p. 1). Essentially, humanity suffers so that it can thrive. Moreover, he states that there are questions that humanity must ask in order to discover this. The questions that should be asked from Wong are as follows [7] (p. 2):

1. What is the meaning of existence? What do we want out of life (aspirations and yearnings)?
2. What does it mean to be true to ourselves (authentic) and find out who we really are (self-identity)?
3. What dualities occur in human existence? How do we find meaning in suffering, pain, and death in order to find personal growth?
4. How are we defined as humanity? War, atrocity, and carnage how do we deal with the psychologically in order to flourish?

The COVID-19 pandemic helped Wong reframe his thinking in terms of PP and EPP, which he felt might still be misunderstood [7]. He noted that during this time of suffering for humanity, the message about suffering to flourish might seem inappropriate. However, Wong postulated that this was incorrect, as the suffering of humanity is inexorable and must be considered if humanity is to flourish. For instance, Wong and Bowers postulate that deep joy can grow during a period of suffering [8]. These authors assert that, as we no longer need to just “survive”, we must find mature human happiness through our suffering in order to overcome the dark side of life and be emotionally authentic. This is particularly apt in developing countries where life is an endless struggle through famine, drought, war, and now the COVID-19 pandemic. Wong asserts that EPP is for everyone but resonates with societies that have an endless fight for survival [7]. He asserts that negative and positive experiences should be integrated, and it is through this struggle that resilience can be built, and psychological growth can occur.

#### 1.1.2. Victor E. Frankl and Existentialism

Frankl, an Austrian Jew, was born in 1905 and died in 1997. He experienced much suffering in his life, although it was varied and rich. After his training as a medical doctor, he went into practice in the realm of psychiatry. He married in 1942; shortly after this, his entire family was sent to concentration camps, where his father, mother, and wife died [9]. He retired from clinical work in 1970 but continued lecturing at various universities, as well as publishing. Frankl received the American Psychiatric Association (APA) award for those who have made significant contributions to psychiatry and religion [10].

Frankl developed logotherapy, which aimed to help people find meaning in life. It helped people understand that, even though they may have experienced suffering and hardship, they must still search for life’s purpose [11]. Essentially, it focuses on the future not the present or past. Frankl also believed that the search for meaning in life is not linked to religiosity or spirituality but what one does or the tasks one fulfils. Moreover, this search for meaning does not have any relation to spirituality or religion but strictly relates to finding purpose in one’s life or tasks [12]. Frankl is a key figure in existential therapy, as he believed that physical and psychological illnesses are disguised “existential angst”. In other words, people have the money and ability to live, but they often have no meaning in their lives. He advocated that people find their purpose in life and, thus, overall meaning through having suffered, worked, and loved [13]. 

Frankl based his therapeutic techniques (logotherapy and existential analysis) on philosophical and psychological notions; he used paradoxical intention, which helps people overcome their anxieties/obsession by using techniques that include self-distancing and humour [14]. He also advocated de-reflection, which means that the therapist moves the person away from what they are obsessing (or hyper-reflecting) about, as too much thought means procrastination and no psychological growth. Another technique, named Socratic dialogue and attitude modification, involves asking a person questions to help them define the meaning in their life. 

Wong clarifies how Frankl’s logotherapy is relevant to the 21st century and to positive psychology [15]. He notes that, in the current technological age, psychology needs rehumanising in terms of finding meaning in life, which endorses Frankl’s initial basic belief; that is, one needs to find a purpose in life, which is consistent with EPP. The following assumptions arising out of Frankl’s beliefs are abbreviated and adapted from Wong [15] (p. 1):

- The self-transcendence hypothesis: Finding meaning in life is the chief motivation in achieving self-transcendence. A person has to do this in order to develop meaning in life as a primary motivation.
- The ultimate meaning hypothesis: This relates to life having an intrinsic meaning and value in any situation. Believing enables the discovery of meaning.
- The meaning mindset hypothesis: A mindset that is linked to success is not as powerful as a “meaning” mindset, which leads to, amongst other things, compassion, happiness, and resilience.
- The freedom of will hypothesis: If one believes in the intrinsic human ability to be responsible and free no matter what, then one is likely to be more independent and genuine (authentic) than those who do not.
- The value hypothesis of discovering meaning: If one is open to being innovative, having new experiences, and having attitudinal values that facilitate wholeness (self-transcendence) as compared to self-interest, one is more likely to find meaning in life. To move from despair and darkness to being able to thrive (flourish).

Wong states that all of these hypotheses are compatible with EPP and emphasise how shifting from self-focus to self-meaning can uplift people [15].

A meaning perspective shifts the focus away from the egotistic pursuit of happiness and success to a compassionate and spiritual worldview of serving a greater good. This shift to the meaning mindset provides a better foundation for positive community mental wellness than the prevalent success/happiness mindset, which encourages cut-throat competition for personal happiness and success. Such pursuits will inevitably lead to discouragement, frustration, and aggression [15] (p. 20).

#### 1.1.3. Molefi Kete Asante and Afrocentricity

Asante (born Arthur Lee Smith Jnr) was born in 1942 [16]. He was involved with the civil rights movement while still in high school, thus embarking on a lifelong process of advocating Black rights [17]. He changed his name, as he considered it an artefact of slavery [18], indicating his disenchantment with the so-called American Dream. He is a pre-eminent figure in African American studies and is still active at Temple University in the United States. One of his chief philosophical contributions is his work on Afrocentricity, which has had a major impact on sociology and psychology.

Afrocentricity’s main argument is that because Eurocentric peoples colonised, enslaved, and dominated people of colour, their culture (western) is not relevant to Africans; indeed, it is completely or diametrically opposed to it [19]. This makes it impossible for Africans to become their true or authentic selves, as African history was negated. 

As a result, Afrocentricity can help Africans reconstruct their culture. In order to do this, Asante asserts that they need to create a collective consciousness [20]. There are many critiques of Afrocentricity, but the foremost one is that it wants to replace or change the Eurocentric view of the world. This is not the case; fundamentally, it seeks to “activate consciousness” in a functional way, which will ensure that Africans see the world through their own lens, through a culture inherent to them so that they can change their lives and, thus, the world [21]. Africans can only find their space or authentic selves if they understand what Afrocentricity means. As a result, Afrocentricity considers the following concepts, adapted from Asante [21] (p. 2) but with our own understandings:

- Any phenomena, to be properly understood, must be located within the psychological time and space of the person taking into account their environment and culture.
- Afrocentricity suggests that all phenomena are dynamic and diverse, so if one is looking at events or experiences from the outside, one must know one’s place in the process.
- The Afrocentric process is a cultural critique that investigates how words are used; for instance, where they originated, how they are used, and how they change over time. As such, actions and behaviours related to words can be analysed as innovative, transformative, or judgemental. This is in order to make sense of events, behaviours, and/or actions.
- Afrocentricity wants to strip the mask of the language of power and privilege to see how stories or myths are established and how they affect the day-to-day life of Africans. This needs reflexion on power, for instance, how it is perceived and predicted.
- Afrocentricity as a process locates political and social power and investigates how it is expressed culturally in terms of, for instance, attitudes, behaviour, and language. This can be individual, institutional, in a text or artefact, or in the location of events (historical and communal).

Afrocentricity is a paradigm based on the idea that African people should re-assert a sense of agency in order to achieve sanity [21] (p. 1). Asante also notes that, through their suffering, they can find true meaning in their lives by engaging in self-reflection, which is compatible with notions in EPP [22].

Bassey asserts that Africana theory is a narrative that makes a critique of the domination of Black people globally [23]. Moreover, it is concerned with concepts that are linked to existence, such as being aware, fear, purposelessness, being desperate, and hopelessness. Africana theory is not a specific philosophy but an umbrella term that reflects critical thinking by Africans and people of African descent [24]. Furthermore, Black existential philosophy is underpinned by the liberation of Black people worldwide from oppression [23]. In this sense, it integrates with the concepts of Afrocentricity. It can be seen that Afrocentricity is a cultural movement aimed at change so that Africans and their descendants (diaspora) are able to find their agency through reflecting on and investigating their development through the historical process [25]. We suggest that they must face their existential anxieties to find meaning in their lives so that they can live authentic lives. Bethea [26] (p. 1) asserts that Black psychology was the precursor of positive psychology. 

The core virtues of positive psychology are very similar to the psychological strengths of African Americans, and they were created in Black psychology before the emergence of positive psychology.

We suggest that African or Black psychology is undeniably linked to Afrocentricity, as it investigates and furthers the overall wellbeing of people of African descent [27]. Bethea states that many of the critical elements of positive psychology are cross-referenced with those of Black psychology; for instance, it looks at how humanity flourishes in spite of any racial differences [26]. Fundamentally, people are resilient in the face of adversity and are able to flourish during adversity. Moreover, Afrocentricity, Africana theory, and Black psychology are linked, as they are all associated with looking at existence, finding meaning, and, ultimately, empowering communities, which, in turn, links them to the theoretical underpinnings of EPP. Afrocentricity, Africana theory, and Black psychology may have theoretical differences, but, fundamentally, they endorse the same thing. They all want African people and those of African descent to be authenticated, that is, to find meaning in their lives and be true to themselves (authentic) so that they will flourish, hence, the link to EPP.

### 1.2. Aligning Wong, Frankl, and Asante in Terms of Concepts of EPP

Wong, Frankl, and Asante all look at the human condition through different lenses, but they all encompass elements of existentialism, positive psychology, and EPP. For instance, Wong notes that life encompasses the ability to be free, to be isolate, and to find life meaningless, which ultimately ends in death [1]. However, to live an authentic life and find one’s self-identity, one must become resilient in order to flourish. A person must ask themselves who they are, why do they exist, and what they need to do in order to find happiness. Humanity as a whole needs to do this in order to prosper—fundamentally, we suffer so that we can understand how to be resilient and find positive change in our lives. In this regard, Frankl wanted to help people define and find the meaning in their lives. In order to do this, they need to overcome any existential anxieties that they have. Fundamentally, they need to find meaning in their life to become their true or authentic selves, which comes through work and suffering. People need to understand that any pain and hardship that they suffer will help them find life’s purpose and, thus, their authentic selves. Moreover, confronting life’s hardships, such as pain, despair, and death, can result in finding meaning and possibilities in life. Asante focuses on Africans and notes that they can only find their authentic selves through examining their life challenges and hardships through looking at the historical adversity that they collectively suffered. To become their true or authentic selves, they need to view the world through Afrocentricity in order to confront the “existential angst” of their history and how this has impacted on their collective culture. We contend that, in doing so, they will find meaning and purpose in life.

It is noted that the opinions and interpretations that we have elucidated are invested in helping people during the COVID-19 pandemic come to terms with life’s existential anxieties (for instance, mortality, isolation, freedom, and life’s meaning). In facing these potential anxieties, people will draw on their strength and resilience in order to find mental wellbeing and to be able to flourish through adversity. 

### 1.3. The COVID-19 Pandemic and How It Has Raised Existential Anxieties and Fears about Not Finding Meaning in Life

COVID-19 has changed the world inexorably. Since the start of the COVID-19 pandemic, mental health globally has worsened for both those with existing disorders [28] and those who have not previously been diagnosed with a psychiatric disorder. Globally, public health officials have expressed concern that fear, related to the pandemic and of the COVID-19 vaccines, could make this challenging situation worse [29]. Psychological illnesses associated with social isolation, anxiety, and fear are on the rise amongst all age-groups [30], which is detrimental to an individual’s overall health and wellbeing [29]. An excessive comorbidity of mental illness in COVID-19 survivors has also been found, which countries must plan for in the short, medium, and long term [30]. This is underpinned by research that reports that exposure to the pandemic and associated stressors has resulted in a rise in mental illnesses related to loneliness and social isolation, such as depression and anxiety [31,32]. This has been found in all population groups globally and puts vulnerable populations at high risk of suicidality [33,34]. Fear and stressors related to contracting the disease and the risk of infection are also associated with the COVID-19 vaccines, which are linked to conspiracy theories on social media [35]. These beliefs have been found in people who display conspiratorial reasoning and some aspects of psychopathology, particularly when they have to deal with external stressors [36]. Individuals with less education are more likely to have these beliefs and are also more likely to show negative attitudes towards governmental actions in addressing COVID-19 challenges [35]. It must be stated that even educated people are prone to believing coronavirus conspiracy myths [37]. There are neurological mechanisms for false beliefs, for instance, neurodegenerative disorders, such as common forms of dementia [38]. Nonetheless, individuals who have normal cognition can also have false beliefs. For instance, those who have difficulty in unpacking scientific theory are more likely to find social media explanations compelling. These individuals are unable to process scientific information in a logical manner and are unlikely to have good problem-solving skills. We assert that all of the aforementioned has led to existential angst globally and more so in developing countries, such as South Africa, where one-third of the population is rural and Black [39], in addition to the fact that 4.4 million Black South Africans are illiterate [40] and an unknown number have a very limited education.

With the aforementioned in mind, making meaning out of life may be difficult during the pandemic, as isolation, financial hardship, and suffering have disturbed the way humanity has traditionally found meaning in their lives [5]. Still, many of us have found different ways to find meaning; for instance, through isolation, we have boosted our resilience and ability to self-reflect, and by self-sacrifice (think of frontline healthcare workers) and caring for others, our suffering has fostered our ability to understand what is meaningful to us.

Wong’s [7] four questions (see Section 1.1.1) help us understand why we exist and, as such, find our true or authentic selves, both individually and collectively. As this is not a research paper, these questions can only be answered generally from a South African perspective in terms of the authors’ situational knowledge. Question 1: South Africa was dominated by slavery, colonialism, separate development, and apartheid, which oppressed the Black majority. For us, the meaning of existence in South Africa is inexorably caught up with this; for all its citizens, we must all be self-aware and take responsibility for our history, and, in that, we can find what we want and need out of life and how we individually and collectively aspire to find meaning. Question 2: South Africans need to become authentic and discover their self-identity by recognising the suffering of the past and taking responsibility for their lives to find out who they really are. Question 3: Improving personal growth in a country in which a large proportion of the population has suffered and still suffers, for instance, in the past and present (COVID-19), affects people of colour more than others, yet they are resilient and flourish through their suffering. Question 4: Psychologically, South Africans are resilient. They are shaped by their past history and present, which is still defined by violence that can be traced back to the oppression of the majority. Forgiveness, anger, and suffering exist, but psychological awareness and resilience allow South Africans to forge ahead to face their existential anxieties and find personal growth in order to flourish and find meaning in life.

### 1.4. How Can EPP Help Heal Existential Anxieties during and after the COVID-19 Pandemic in South Africa

Both individually and collectively, the COVID-19 pandemic has revealed existential concerns and elicited significant anxiety in the global population. One of the main problems of the COVID-19 pandemic in South Africa is isolation due to lockdowns instituted by the government in order to halt the progression of the disease, which resulted in being physically distanced from others. This has led to a narrowing of social circles and caused distress for many [41]. In some instances, the need to belong has led to defiance of compliance with COVID-19 regulations, and many people have continued to engage in close social interaction with others, often leading to an increased spread of the virus in communities [42]. This would have led them to experience increased anxiety.

Furthermore, for many, the realisation of existential freedom has been challenging, especially with decisions about whether to travel or visit family members or friends and still stay safe. Many have been conflicted and unsure of how to navigate an unpredictable world where their decisions have significant consequences for themselves and the lives of others. These changes have also led to many people wrestling with their self-identity. For example, many people in South Africa have lost their jobs due to the COVID-19 pandemic, causing them to feel threatened economically, as well as existentially (as they no longer recognise or know themselves). 

Thus, the COVID-19 pandemic has emphasised our existential angst, and, at times, all of us have felt stress, anxiety, and fear of death, while many of us have become (and still are) depressed. Existential positive psychology (EPP) is an approach that helps people who want to lead authentic lives face adversity in order to flourish. The world will not be the same again; there is what some call a “new normality”. Humanity does not fully trust itself and has not yet learnt to adapt to the overwhelming reality of the pandemic, but it has started to “hope” again. This is in spite of the increase in levels of loneliness and depression in many countries [2]. The pandemic has caused a “perfect storm” in terms of mental health and wellness. Human suffering is existential in nature, but therapists and other health professionals can be taught to help people see that feelings of hopelessness can be superseded by enthusiasm for life using interventions based on EPP [2,43,44]. This can also happen in times of death and dying. These moments, especially with the support and acceptance of loved ones, can be joyous, as we are released from the human condition, because through suffering, trauma, and adversity, humanity flourishes [45] and becomes resilient. A model formulated on EPPs’ model of suffering can also be applied during the global pandemic, and it could be a useful clinical tool in South Africa and other countries. The model has three notions: firstly, that through suffering existential concerns are revealed during the pandemic; secondly, that existential anxiety damages humanity’s capacity to find meaning in life; and thirdly, that nurturing meaning will alleviate existential anxiety, which will allow human growth and facilitate flourishing. The model can be used by practitioners with those who are suffering during the COVID-19 pandemic by integrating the theory into applied clinical work [46]. 

Wong, Frankl, and Asante’s philosophical foundations are all linked to the premise that reflections and the acknowledgement of suffering or adversity, whether it is in living memory or historical, are needed if humanity (all ethnicities) can prosper in the future. Furthermore, to acknowledge that life and death are a continuum and accept responsibility for how one deals with this in order to find our authentic and true selves’, humanity has to play both the “black and white keys” [2]. Although Wong and his co-authors were referring to the dark and light sides of humanity’s existential angst, we feel that their analogy is extraordinarily appropriate. 

The majority of South Africans have suffered, but this can be redemptive, as through resilience, they have been able to connect their lives to “spiritual dimensions and values” [2] and their ancestral heritage. Humanity must individually and collectively find itself and look for meaning. We need to find existential courage so that our mindset is positive. We must be able to transcend as individuals and as a collective; otherwise, it is unlikely that South Africa (or Gaia) will be a healthy and happy environment. Recent findings suggest that resilience and affective balance are important aspects of implementing meaning-based preventions and interventions using EPP [47]. Given the importance of meaning in the coping process of adverse experiences due to the COVID-19 pandemic, mental health providers could use meaning-based strategies to foster the promotion of mental health and reduce the risk of psychological challenges. Existential positive psychology will help people build resilience and flourish during the pandemic, because it expands their understanding of suffering in a way that will help them transform their lives [2]. 

## 2. Conclusions

It is apparent that the clinical implications of EPP lie at the intersection of theory and practice. This paper highlights that EPP is a positive tool that can be used by clinicians in South Africa, or globally, in order to deal with their client’s anxieties and mental health challenges due to the COVID-19 pandemic. The theoretical underpinnings of EPP contend that mental health concern is an existential issue that gives rise to anxiety, which erodes meaning and, thus, can impair psychological functioning. The use of EPP as a positive tool can enhance resilience and overall mental wellness in clients that experience mental health problems, allowing them to flourish. It is a buffer against the existential anxiety engendered by considering life’s deepest realities. Wong, Frankl, and Asante, we assert, underpin areas that should be reflected on when using EPP in both the theoretical and practical realms. We hope that future clinical research will continue to advance work in this exciting area of enquiry and practice.

## Data Availability

Not applicable.
